# Survival Benefit of Crossover Administration of Regorafenib and Trifluridine/Tipiracil Hydrochloride for Patients With Metastatic Colorectal Cancer: Exploratory Analysis of a Japanese Society for Cancer of the Colon and Rectum Multicenter Observational Study (REGOTAS)

**DOI:** 10.3389/fonc.2021.576036

**Published:** 2021-03-08

**Authors:** Keigo Chida, Daisuke Kotani, Toshikazu Moriwaki, Shota Fukuoka, Toshiki Masuishi, Atsuo Takashima, Yosuke Kumekawa, Takeshi Kajiwara, Kentaro Yamazaki, Masato Komoda, Akitaka Makiyama, Tadamichi Denda, Yukimasa Hatachi, Takeshi Suto, Naotoshi Sugimoto, Masanobu Enomoto, Toshiaki Ishikawa, Tomomi Kashiwada, Koji Ando, Satoshi Yuki, Yoshihiro Okita, Hitoshi Kusaba, Daisuke Sakai, Koichi Okamoto, Takao Tamura, Kimihiro Yamashita, Masahiko Gosho, Yasuhiro Shimada

**Affiliations:** ^1^Department of Gastrointestinal Oncology, National Cancer Center Hospital East, Kashiwa, Japan; ^2^Division of Gastroenterology, Faculty of Medicine, University of Tsukuba, Tsukuba, Japan; ^3^Department of Clinical Oncology, Aichi Cancer Center Hospital, Aichi, Japan; ^4^Gastrointestinal Medical Oncology Division, National Cancer Center Hospital, Tokyo, Japan; ^5^Department of Gastroenterology, Saitama Cancer Center, Saitama, Japan; ^6^Department of Gastrointestinal Medical Oncology, National Hospital Organization Shikoku Cancer Center, Ehime, Japan; ^7^Division of Gastrointestinal Oncology, Shizuoka Cancer Center, Shizuoka, Japan; ^8^Department of Gastrointestinal and Medical Oncology, National Hospital Organization Kyushu Cancer Center, Fukuoka, Japan; ^9^Department of Hematology/Oncology, Japan Community Healthcare Organization Kyushu Hospital, Fukuoka, Japan; ^10^Cancer Center, Gifu University Hospital, Gifu, Japan; ^11^Division of Gastroenterology, Chiba Cancer Center, Chiba, Japan; ^12^Department of Medical Oncology, Kobe City Medical Center General Hospital, Hyogo, Japan; ^13^Department of Gastroenterological Surgery, Yamagata Prefectural Central Hospital, Yamagata, Japan; ^14^Department of Medical Oncology, Osaka International Cancer Institute, Osaka, Japan; ^15^Department of Gastrointestinal and Pediatric Surgery, Tokyo Medical University, Tokyo, Japan; ^16^Department of Specialized Surgeries, Graduate School of Medicine and Dentistry, Tokyo Medical and Dental University, Tokyo, Japan; ^17^Division of Hematology, Respiratory Medicine and Oncology, Department of Internal Medicine, Faculty of Medicine, Saga University, Saga, Japan; ^18^Department Surgery and Science, Graduate School of Medical Sciences, Kyushu University, Fukuoka, Japan; ^19^Department of Cancer Chemotherapy, Hokkaido University Hospital Cancer Center, Sapporo, Japan; ^20^Department of Clinical Oncology, Faculty of Medicine, Kagawa University, Kagawa, Japan; ^21^Department of Medicine and Comprehensive Biosystemic Science, Kyushu University Graduate of Medical Sciences, Fukuoka, Japan; ^22^Department of Frontier Science for Cancer and Chemotherapy, Osaka University Graduate School of Medicine, Osaka, Japan; ^23^Department of Surgery, National Defense Medical College, Tokorozawa, Japan; ^24^Department of Medical Oncology, Faculty of Medicine, Kindai University, Osaka, Japan; ^25^Division of Gastrointestinal Surgery, Department of Surgery, Graduate School of Medicine, Kobe University, Hyogo, Japan; ^26^Department of Biostatistics, Faculty of Medicine, University of Tsukuba, Tsukuba, Japan; ^27^Clinical Oncology Division, Kochi Health Sciences Center, Kochi, Japan

**Keywords:** regorafenib, trifluridine/tipiracil hydrochloride, colorectal cancer, prognosis, chemotherapy – oncology

## Abstract

**Background:** The survival benefits of regorafenib (REG) and trifluridine/tipiracil hydrochloride (TFTD) have been demonstrated in chemorefractory patients with metastatic colorectal cancer (mCRC). However, the effects of crossover administration of REG and TFTD on patient survival remain unclear. The present study evaluated the association between exposure to REG and TFTD and overall survival (OS) in patients with mCRC using data from the REGOTAS study.

**Patients and Methods:** We analyzed patients registered in the REGOTAS study, which retrospectively compared the efficacy and safety of use of REG or TFTD as later-line chemotherapy for chemorefractory mCRC patients. We compared the survival outcomes of cohort A (treated using both REG and TFTD) and cohort B (treated using either REG or TFTD).

**Results:** A total of 550 patients (cohort A, *n* = 252; cohort B, *n* = 298) met the inclusion criteria. The median OS was significantly increased in cohort A compared with cohort B [9.6 months (95% confidence interval (CI), 8.9–10.9 months) vs. 5.2 months (95% CI, 4.4–6.0 months), *P* < 0.001]. Multivariate analysis revealed that cohort A was independently associated with a significant increase in OS [A vs. B: Hazard ratios (HR), 0.58; 95% CI, 0.47–0.72; *P* < 0.001]. Subgroup analysis adjusted using multivariate Cox model revealed a consistently better trend in most subgroups for cohort A compared with cohort B.

**Conclusions:** Our study revealed prolonged survival in patients treated with REG and TFTD. Therefore, all active agents, including REG and TFTD, should be made available to mCRC patients.

## Introduction

Colorectal cancer (CRC) is one of the leading causes of cancer-related deaths worldwide (1). The development of combination chemotherapy regimens involving cytotoxic agents [such as fluoropyrimidine (FU), oxaliplatin (OX), and irinotecan (IRI)] and molecular targeted therapies (such as bevacizumab, ramucirumab, ziv-aflibercept, cetuximab, and panitumumab) has increased the survival of metastatic CRC (mCRC) patients by around 30 months (2–8). In the CORRECT and RECOURSE phase III trials, the active agents, regorafenib (REG) and trifluridine/tipiracil hydrochloride (TFTD), significantly improved overall survival (OS) in patients with chemorefractory mCRC (9, 10).

The strategic availability of the active ingredients FU, OX, and IRI for all mCRC patients suitable for systemic chemotherapy maximizes OS (11). However, there are few reports of the benefits of using both REG and TFTD as a salvage therapy to improve OS in mCRC patients (12).

We previously reported the REGOTAS study, which was a multicenter, large cohort, observational study, showed no significant difference in OS between treatment using REG and TFTD in patients with mCRC. The present study compared patients treated using both REG and TFTD with those treated with either REG or TFTD alone in the REGOTAS study to assess the effects of exposure to REG and TFTD on OS in patients with mCRC who received FU, OX, IRI, and bevacizumab, as well as anti-EGFR antibody (in patients with wild type *KRAS*/*NRAS* tumors).

## Methods

### Patients

The present study retrospectively examined the clinical records of patients with mCRC treated with later-line chemotherapy comprising REG or TFTD during the period from June 1, 2014 to November 30, 2015 in the participating institutions. All patients were registered in the REGOTAS study, which is described in detail elsewhere (13). The main eligibility criteria were: (1) histologically confirmed colorectal adenocarcinoma; (2) no prior treatment using REG and TFTD; (3) previous treatment with FU, OX, IRI, bevacizumab, and anti-EGFR antibody (in patients with wild type *KRAS*/*NRAS* tumors); (4) Eastern Cooperative Oncology Group performance status (ECOG PS) of 0–2; and (5) adequate organ function. The present study was approved by the ethics committees at each institution and was in accordance with the guidelines for biomedical research specified in the Declaration of Helsinki. The REGOTAS study was registered with the University Medical Information Network (number UMIN000020416). The requirement for informed consent was waived due to the retrospective design of this study.

### Statistical Analysis

The exploratory primary endpoint was OS of all patients stratified by exposure to REG and/or TFTD as follows: cohort A (both REG and TFTD) and cohort B (either REG or TFTD). The following pretreatment clinical data and baseline laboratory values were used in the analysis as covariates: age, sex, body mass index, ECOG PS, primary tumor site, surgery on primary tumor, RAS status, metastatic tumor site (liver metastasis, lung metastasis, lymph node metastasis, peritoneal dissemination, and bone metastasis), number of metastatic sites, pathologic type, time from initiation of first-line chemotherapy to initiation of later-line treatment, serum albumin, serum aspartate transaminase (AST), serum C-reactive protein (CRP), and serum carcinoembryonic antigen (CEA). Each cutoff value of quantitative data was set with reference to that of albumin, AST, CRP, AST, and CEA in the REGOTAS study (13).

OS was defined as the time from the start of initial REG or TFTD to death or last follow-up. Quantitative data are expressed as median and interquartile range (IQR). The Mann–Whitney *U*-test was used to compare the continuous variables, and Fisher's exact test was performed to compare the categorical variables. Survival curves were estimated using the Kaplan–Meier method, and differences between the groups were tested by the log-rank test. Hazard ratios (HRs) were estimated using the Cox proportional hazard model. OS was analyzed using univariate and multivariate Cox regression analyses. The backward selection method was conducted for the selection of factors retained (*P* < 0.2) in the multivariate analysis. The predictive factor for OS between each group was explored using subgroup analyses with the multivariate Cox model including interaction terms.

A 1:1 matching using the propensity score (propensity score-matched dataset) was performed as a sensitivity analysis. Patients in the two groups were matched by a difference of propensity score within 0.05. The propensity score was calculated with a multivariate logistic regression model including 19 prognostic variables (Supplementary Table 1). All *P*-values < 0.05 were considered statistically significant. All statistical analyses were performed using EZR (Saitama Medical Center, Jichi Medical University, Saitama, Japan), which is a graphical user interface for R (The R Foundation for Statistical Computing, Vienna, Austria).

## Results

### Patients

**Figure 1:**
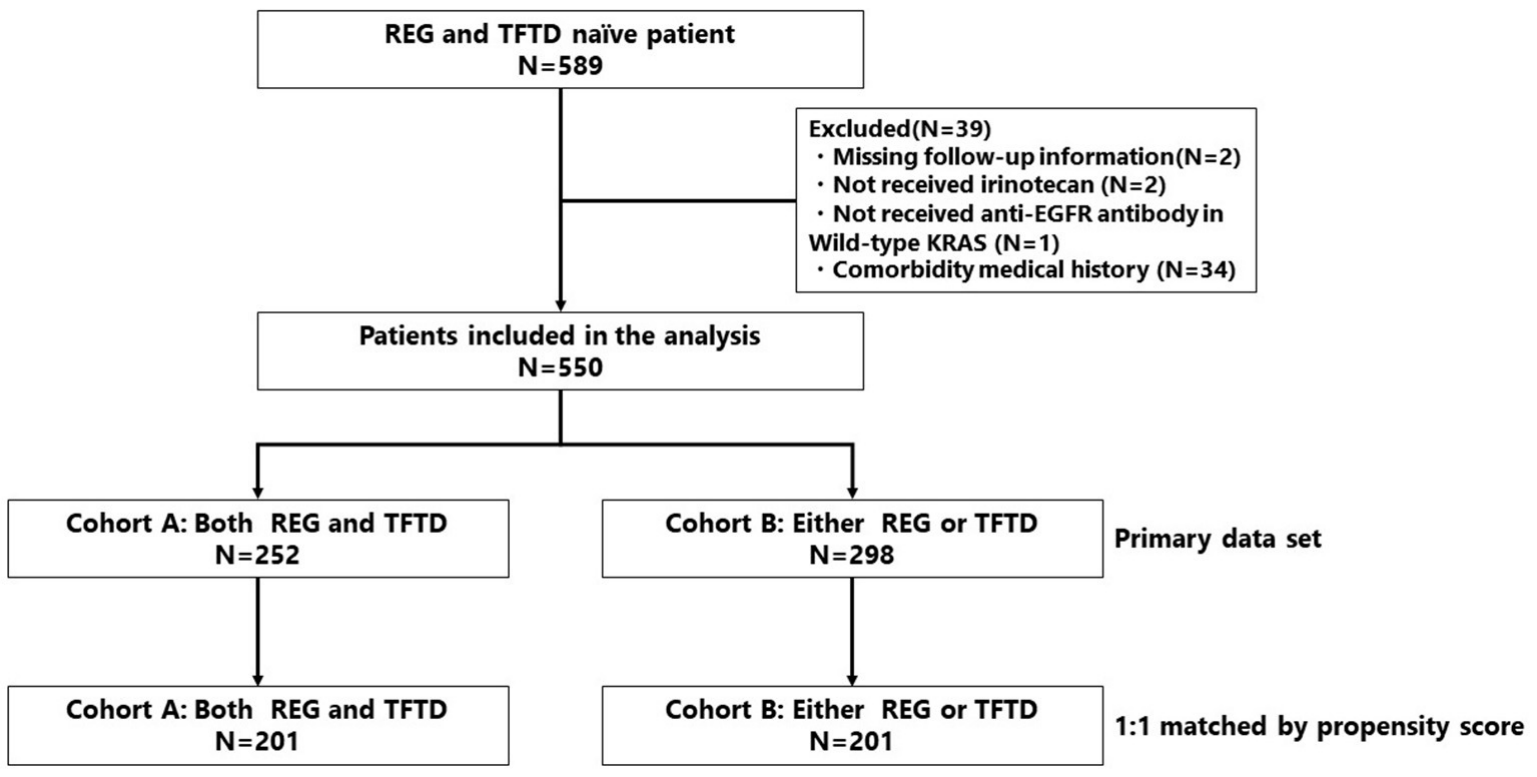
Patient selection flow diagram.

Among 589 mCRC patients, 550 met the inclusion criteria (cohort A, *n* = 252; cohort B, *n* = 298) (Figure 1). The patient characteristics are summarized in Table 1. Significant differences between cohorts A and B were found for the following factors: ECOG PS 0 (49 vs. 33%, respectively; *P* < 0.001), lymph node metastasis (37 vs. 48%, respectively; *P* = 0.012), peritoneal dissemination (12 vs. 24%, respectively; *P* < 0.001), number of metastatic organ sites ≥ 2 (71 vs. 79%, respectively; *P* = 0.007), baseline serum albumin <3.5 g/dL (29 vs. 58%, respectively; *P* < 0.001), baseline serum AST ≥40 IU/L (20 vs. 42%, respectively; *P* < 0.001), and baseline serum CRP ≥1 mg/dL (34 vs. 52%, respectively; *P* < 0.001). All patients received FU, OX, IRI, and bevacizumab, and all patients with wild type *KRAS*/*NRAS* tumors received anti-EGFR antibody.

**Table 1:** Patient characteristics.

	**cohort A (*****n*** **= 252)**		**cohort B (*****n*** **= 298)**		*P*-value^*^
**Age, year**					
Median (IQR)	65	(57–71)	64	(54–69)	0.066
≥65 years, *n* (%)	110	(44)	153	(51)	0.087
**Sex**, ***n*** **(%)**					0.543
Male	144	(57)	179	(60)	
Female	108	(43)	119	(40)	
**Body mass index**, ***n*** **(%)**					0.417
<18.5 kg/m^2^	37	(17)	52	(17)	
≥18.5 kg/m^2^	215	(83)	246	(83)	
**ECOG PS**, ***n*** **(%)**					<0.001
0	124	(49)	99	(33)	
1 or 2	128	(51)	199	(67)	
**Primary tumor site**, ***n*** **(%)**					0.758
Right	54	(21)	68	(23)	
Left	198	(79)	230	(77)	
**Surgery on primary tumor site**, ***n*** **(%)**					
Yes	45	(18)	74	(25)	0.061
**Pathologic type**, ***n*** **(%)**					0.334
Well-moderately differentiated adenocarcinoma	226	(90)	268	(90)	
Others	14	(5)	22	(7)	
Missing data	12	(5)	8	(3)	
***RAS*** **status**, ***n*** **(%)**					0.261
WT	131	(52)	137	(46)	
MT	118	(47)	152	(51)	
Missing data	3	(1)	9	(3)	
**Metastatic organ site**, ***n*** **(%)**					
Liver	158	(63)	184	(62)	0.860
Lung	168	(67)	195	(65)	0.787
Lymph node	94	(37)	143	(48)	0.012
Peritoneal dissemination	30	(12)	72	(24)	<0.001
Bone	17	(7)	36	(12)	0.049
**Number of metastatic organ site(s)**, ***n*** **(%)**					0.055
1	72	(29)	63	(21)	
≥2	180	(71)	235	(79)	
**Drug exposure**, ***n*** **(%)**					1.000
Fluoropyrimidine	252	(100)	298	(100)	
Oxaliplatin	252	(100)	298	(100)	
Irinotecan	252	(100)	298	(100)	
Bevacizumab	252	(100)	298	(100)	
Anti-EGFR antibody (in patients with wild type *KRAS/NRAS* tumors)	131	(100)	137	(100)	
**Intolerable drug**, ***n*** **(%)**					
Any drugs	79	(31)	96	(32)	0.900
Fluoropyrimidine	4	(2)	22	(7)	0.003
Oxaliplatin	66	(26)	77	(26)	1.000
Irinotecan	8	(3)	28	(9)	0.006
Bevacizumab	11	(4)	31	(10)	0.013
Anti-EGFR antibody (in patients with wild type *KRAS/NRAS* tumors)	5	(2)	10	(3)	0.471
**Prior regimens**, ***n*** **(%)**					0.892
≥3	125	(50)	145	(49)	
**Time since initiation of first-line chemotherapy**, ***n*** **(%)**					0.152
<18 months	59	(23)	87	(29)	
≥18 months	193	(77)	211	(71)	
**Baseline albumin**, ***n*** **(%)**					
<3.5 g/dL	72	(29)	172	(58)	<0.001
Missing	6	(2)	9	(3)	
**Baseline serum AST**, ***n*** **(%)**					
≥40 IU/L	50	(20)	107	(36)	<0.001
Missing data	2	(1)	1	(0.5)	
**Baseline CRP**, ***n*** **(%)**					
≥1 mg/dL	85	(34)	156	(52)	<0.001
Missing data	7	(3)	9	(3)	
**Baseline serum CEA**, ***n*** **(%)**					
≥5 ng/mL	218	(87)	270	(91)	0.253
Missing data	3	(1)	4	(1)	

* *P-values were calculated by Fisher's exact probability test for categorical variables*.

*IQR, interquartile range; EGFR, epidermal growth factor receptor; RAS, rat sarcoma; AST, aspartate transaminase; CEA, carcinoembryonic antigen; WT, wild-type; MT, mutant*.

### Efficacy

**Figure 2:**
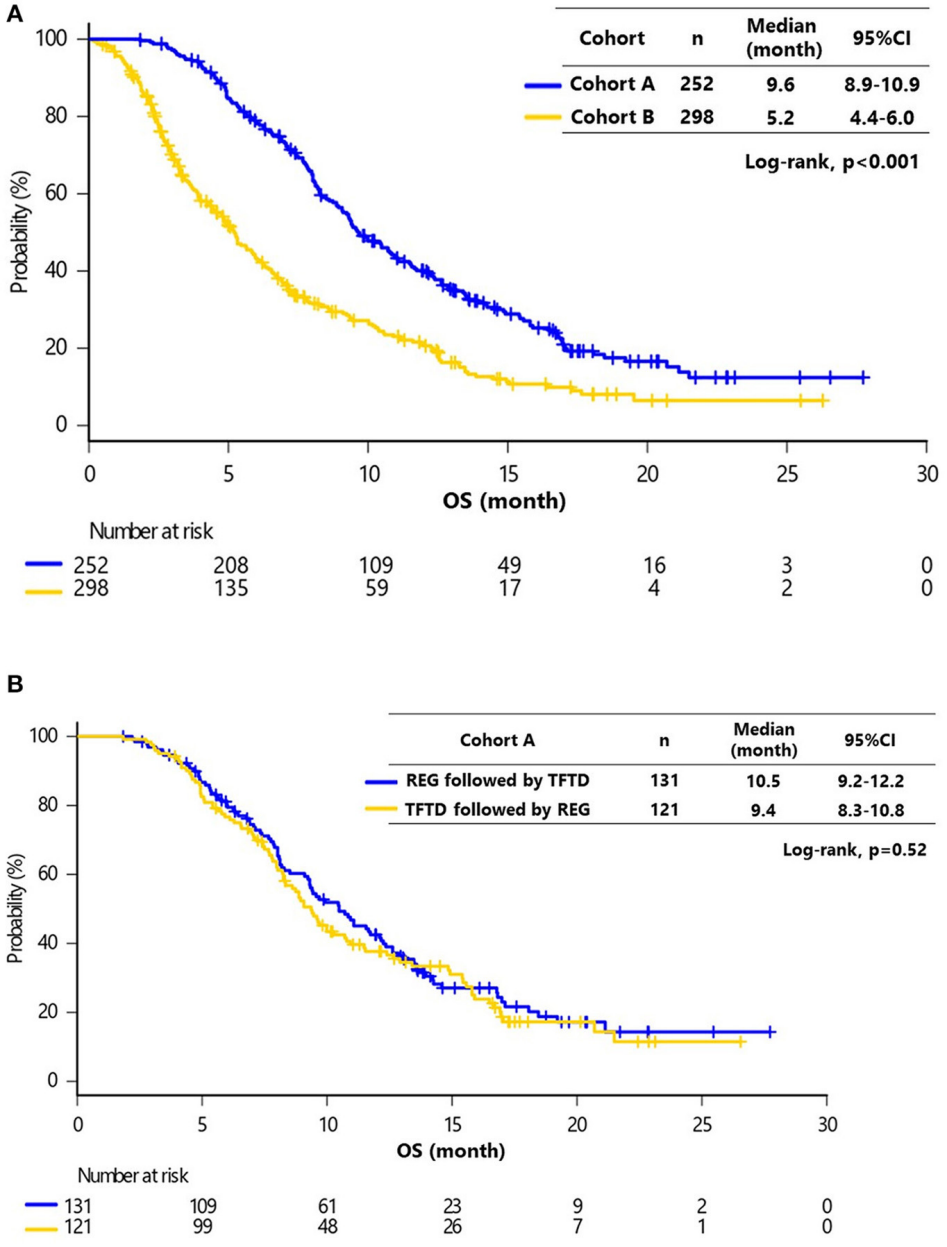
**(A)** Kaplan–Meier curves of overall survival (OS) in cohorts A and B. The median OS of cohorts A and B were 9.6 months (95% CI, 8.9–10.9) and 5.2 months (95% CI, 4.4–6.0), respectively (Log-rank, *P* < 0.001). **(B)** Kaplan–Meier curves of OS in cohort A (REG followed by TFTD vs. TFTD followed by REG). The median OS values of REG followed by TFTD and TFTD followed by REG were 10.5 months (95% CI, 10.2–16.1) and 9.4 months (95% CI, 7.3–17.3), respectively (Log-rank, *P* = 0.52).

The median follow-up at the time of analysis was 17.3 months [95% confidence interval (CI), 16.1–18.0 months]. The median OS for all patients was 6.8 months (95% CI, 3.4–11.5 months), and 418 (76%) patients had died. The median follow-up was significantly longer in cohort A compared with cohort B (17.6 vs. 15.2 months, respectively; *P* < 0.001). The median OS was significantly greater in cohort A compared with cohort B [9.6 (95% CI, 8.9–10.9) months vs. 5.2 months (95% CI, 4.4–6.0 months), respectively; *P* < 0.001] (Figure 2A). There was no significant difference in OS between patients receiving REG followed by TFTD and TFTD followed by REG in cohort A [10.5 months (95% CI, 9.2–12.2 months) vs. 9.4 months (95% CI, 8.3–10.8 months), *P* = 0.52] (Figure 2B).

Table 2 shows the results of univariate and multivariate analyses for OS. In these analyses, the factors significantly associated with OS were cohort (A vs. B: HR, 0.58; 95% CI, 0.47–0.72; *P* < 0.001), ECOG PS (1–2 vs. 0: HR, 1.44; 95% CI, 1.16–1.78; *P* = 0.001), albumin (≥3.5 vs. <3.5 g/dL: HR, 0.77; 95% CI, 0.63–0.94; *P* = 0.012); AST (≥40 vs. <40 IU/L: HR, 1.48; 95% CI, 1.24–1.76; *P* < 0.001), CRP (≥1.0 vs. <1.0 mg/dL: HR, 1.84; 95% CI, 1.46–2.32; *P* < 0.001), CEA (≥ 5 vs. <5 ng/mL: HR, 1.69; 95% CI, 1.16–2.47; *P* = 0.006), liver metastasis (Yes vs. No: HR, 1.51; 95% CI, 1.17–1.94; *P* = 0.001), peritoneal dissemination (Yes vs. No: HR, 1.38; 95% CI, 1.05–1.82; *P* = 0.023), and time since initiation of first-line chemotherapy (≥18 vs. <18 months: HR, 0.64; 95% CI, 0.50–0.81; *P* < 0.001).

**Table 2:** Univariate and multivariate analyses of overall survival (OS).

		**Univariate**				**Multivariate**			
**Variable**	**Category**	**HR**	**Lower**	**Upper**	*P*-value^*^	**HR**	**Lower**	**Upper**	*P*-value^*^
Treatment	Cohort A vs. cohort B	0.48	0.40	0.59	<0.001	0.58	0.47	0.72	<0.001
Age	≥65 vs. <65	1.22	1.01	1.48	0.044	1.17	0.96	1.43	0.127
Gender	Male vs. Female	0.95	0.79	1.16	0.634				
Body mass index	<18.5 kg/m^2^ vs. ≥18.5	0.94	0.72	1.22	0.623				
ECOG PS	1 or 2 vs. 0	1.64	1.35	2.01	<0.001	1.44	1.16	1.78	0.001
Primary tumor site	Left vs. Right	0.79	0.63	0.99	0.042	0.88	0.69	1.13	0.315
Surgery on primary tumor site	Yes vs. No	0.60	0.48	0.76	<0.001	0.80	0.62	1.02	0.071
Pathologic type	Well-moderately differentiated vs. others	0.97	0.64	1.46	0.874				
Baseline serum albumin	>3.5 g/dL vs. <3.5	0.51	0.43	0.62	<0.001	0.77	0.63	0.94	0.012
Baseline serum AST	≥40 IU/L vs. <40	1.84	1.53	2.22	<0.001	1.48	1.24	1.76	<0.001
Baseline serum CRP	≥1.0 mg/dL vs. <1.0	1.68	1.44	1.95	<0.001	1.84	1.46	2.32	<0.001
Baseline serum CEA	≥5 ng/mL vs. <5	1.85	1.38	2.49	<0.001	1.69	1.16	2.47	0.006
Liver metastasis	Yes vs. No	1.65	1.35	2.03	<0.001	1.51	1.17	1.94	0.001
Lung metastasis	Yes vs. No	0.84	0.69	1.03	0.089	0.95	0.75	1.49	0.691
Lymph node metastasis	Yes vs. No	1.40	1.15	1.70	<0.001	1.18	0.94	1.49	0.150
Peritoneal dissemination	Yes vs. No	1.52	1.20	1.93	<0.001	1.38	1.05	1.82	0.023
Bone	Yes vs. No	0.96	0.70	1.34	0.829				
Number of metastatic organ sites	≥2 vs. 1	1.48	1.18	1.87	0.001	1.05	0.76	1.43	0.777
Prior regimens	≥3 *vs*. <3	0.85	0.70	1.04	0.108	0.79	0.61	1.03	0.078
Time since initiation of first-line chemotherapy	≥18 months vs. <18	0.63	0.51	0.78	<0.001	0.64	0.50	0.81	<0.001
*RAS* status	MT vs. WT	1.18	0.99	1.41	0.067	0.91	0.71	1.16	0.455

* *P-values were calculated using the Cox proportional-hazards model*.

*RAS, rat sarcoma; AST, aspartate transaminase; CEA, carcinoembryonic antigen; WT, wild-type; MT, mutant*.

**Figure 3:**
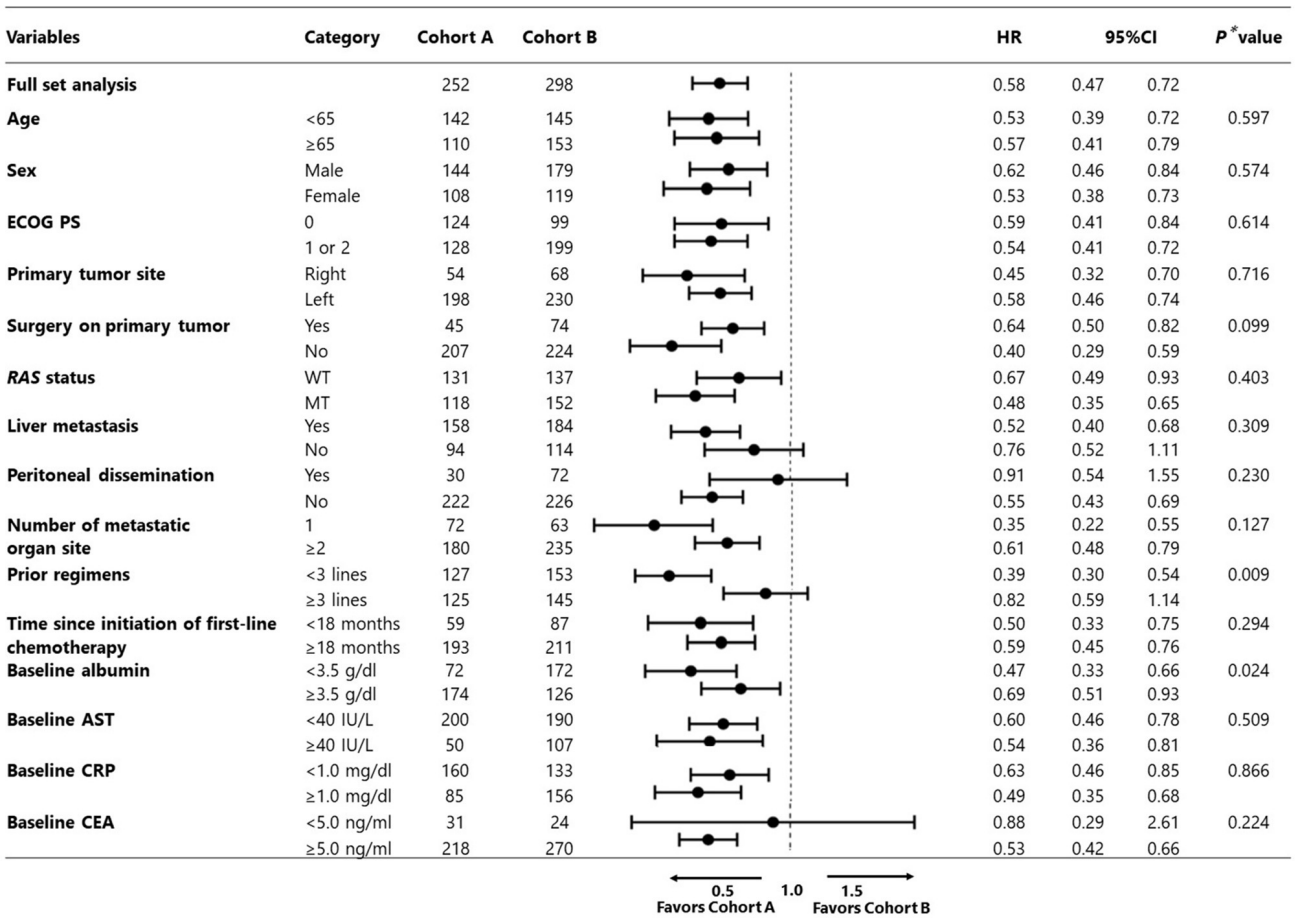
Subgroup analysis adjusted by multivariate Cox model (cohort A vs. B).

In the subgroup analysis adjusted using the multivariate Cox model, cohort A demonstrated consistently better trends in almost all subgroups examined compared with cohort B (Figure 3).

### Safety and Toxicity

Safety and toxicity are shown in Table 3. There was no significant difference in incidence of grade ≥3 hematologic toxicities between cohorts A and B, except for anemia (5 vs. 11%, respectively; *P* = 0.019). Additionally, for nonhematologic toxicities, incidence of grade ≥3 anorexia was higher in cohort B than cohort A (2 vs. 8%, respectively; *P* = 0.001), whereas the incidence of hand–foot skin reaction was higher in cohort A than cohort B (13 vs. 4%, respectively; *P* < 0.001).

**Table 3:** Frequency of treatment-related grade ≥3 adverse events (AE).

**Variable**	**cohort A (*****n*** **= 252)**		**cohort B (*****n*** **= 298)**		*P*-value^*^
**Hematologic toxicities**, ***n*** **(%)**					
Any	67	(27)	91	(31)	0.355
Neutropenia	50	(20)	63	(21)	0.674
Anemia	13	(5)	33	(11)	0.019
Thrombocytopenia	12	(5)	13	(4)	0.985
**Non-hematologic toxicities**, ***n*** **(%)**					
Any	65	(26)	80	(27)	0.856
Fatigue	3	(1)	12	(4)	0.076
Anorexia	4	(2)	24	(8)	0.001
Febrile neutropenia	3	(1)	6	(2)	0.674
Hand-foot skin reaction	32	(13)	12	(4)	<0.001
Liver dysfunction	14	(6)	14	(5)	0.794

* *P-values were calculated by Fisher's exact probability test for categorical variables*.

*AE, adverse event*.

### Sensitivity Analysis

A total of 201 patients per group were matched by propensity score. Patients' characteristics were well-balanced between the two groups (Supplementary Table 2), and the median OS was found to be significantly longer in cohort A compared with that of cohort B [9.3 months (95% CI, 8.2–10.5 months) vs. 5.3 months (95% CI, 4.8–6.7 months), *P* < 0.001] as in the observational dataset (Supplementary Figure 1). The incidence of grade ≥3 toxicity was also similar to that in the observational dataset, except for the incidence of hand–foot skin reaction (Supplementary Table 3).

## Discussion

To the best of our knowledge, this is the first study to report the beneficial effects of the crossover administration of REG and TFTD on survival of patients with chemorefractory mCRC. Although increased exposure to standard chemotherapy agents, such as FU, OX, and IRI, and molecular targeting agents, including bevacizumab and anti-EGFR antibodies, contribute to a prolongation of OS (11, 14), our findings suggested that making all key active agents, including REG and TFTD, available could further improve OS in mCRC patients.

It has not previously been shown that treatment with both REG and TFTD contributes to longer OS compared with use of either REG or TFTD alone in patients with chemorefractory mCRC. The CORRECT trial did not include patients who had previously received TFTD, and the RECOURSE trial included only 18% of patients who had previously received REG (9, 10). Furthermore, details of post-study treatments were not reported in either of these phase III studies. Our findings indicated that treatment with both REG and TFTD improved OS compared with use of either REG or TFTD alone in mCRC patients who were refractory or intolerant to standard chemotherapy, irrespective of the subgroup. While the optimal sequential order of REG and TFTD therapy remains unclear, there was no significant difference in OS between the patients in cohort A who received TFTD followed by REG or REG followed by TFTD. These data support the findings from an Italian retrospective study in which patients received both REG and TFTD that showed that the median OS was 12.8 months (95% CI, 10.2–14.4 months) for REG followed by TFTD and 10.3 months (95% CI, 8.7–14.4 months) for TFTD followed by REG (15).

The differences observed in patients' characteristics indicate the requirement for both REG and TFTD to be made available to patients with chemorefractory mCRC. In the present study, patients with ECOG PS 1 or 2, peritoneal dissemination, albumin <3.5 g/dL, AST ≥40 IU/L, or CRP ≥1 mg/dL had fewer chances to receive both REG and TFTD. The consistent efficacy of crossover administration of REG and TFTD irrespective of the subgroup highlights that exposure to both REG and TFTD contributes to improved OS in patients with poor prognostic factors.

It is important to note that the nature of this analysis may have led to an inherent bias. In particular, patients who live longer have a greater opportunity to be treated with more lines of chemotherapies. Furthermore, patients with poor ECOG PS or a shorter life expectancy may have been excluded from receiving the salvage-line chemotherapy of REG and/or TFTD. Therefore, we only analyzed patients who were refractory or intolerant to standard chemotherapies [FU, OX, IRI, bevacizumab, and anti-EGFR antibody (if the patients had wild type *KRAS*/ *NRAS* tumor)] in order to minimalize the inherent bias. Multivariate analysis of prognostic factors also demonstrated that crossover administration of REG and TFTD was independently associated with significant OS prolongation. In addition, subgroup analysis adjusted by the multivariate Cox model revealed that cohort A consistently demonstrated better trends in almost all subgroups examined compared with cohort B. These findings highlight the importance of making active agents, including REG and TFTD, available to all patients.

However, while making these active agents available is a valuable treatment strategy, the OS of these patients remains unsatisfactory and warrants further improvement. A promising efficacy of TFTD with bevacizumab was previously reported in a phase I–II trial (C-task force) (16), and was recently replicated in retrospective and prospective studies (17, 18). In addition, the combination of REG with nivolumab showed manageable toxicities and encouraging antitumor activity in microsatellite stable mCRC patients (19). We believe that these combination therapies are effective strategies to prolong OS in patients with chemorefractory mCRC.

The present study had some limitations that should be considered when interpreting the results. First, this was a non-randomized retrospective study with a limited sample size. Second, all patients enrolled in this study were Japanese. However, the absence of ethnic differences in the analysis of the efficacy of REG and TFTD in the phase III trials could enable the results to be applied to all patients, regardless of ethnicity (9, 10, 20, 21).

## Conclusions

Our multicenter retrospective study revealed the survival benefits of crossover administration of REG and TFTD. Our findings highlight the importance of making all active agents, including REG and TFTD, available to patients with mCRC.

## Data Availability Statement

The raw data supporting the conclusions of this article will be made available by the authors, without undue reservation, to any qualified researcher.

## Ethics Statement

The studies involving human participants were reviewed and approved by National Cancer Center Hospital East and each participating facilities. Written informed consent for participation was not required for this study in accordance with the national legislation and the institutional requirements.

## Author Contributions

KC, DK, and YS: conception and design and development of methodology. SF, TMo, AT, YK, TKaj, KYamaz, MK, AM, TD, YH, TS, NS, ME, TI, TKas, KA, SY, YO, HK, DS, KO, TT, and KYamas: acquisition of data. KC, DK, TMo, and MG: analysis and interpretation of data. KC, DK, and TMo: writing, review and/or revision of the manuscript. TMo and YS: study supervision. All authors contributed to the article and approved the submitted version.

## Conflict of Interest

DK received honoraria from Takeda, Chugai, Lilly, and Merck Serono. TMo received a research grant from Chugai Pharma, Takeda, Taiho Pharmaceutical, Yakult Honsha Boehringer Ingelheim, Merck Sharp and Dohme Oncology, Sanofi Aventis. TMa has received honoraria from Takeda, Chugai, Merck Serono, Taiho, Bayer, Lilly Japan, Yakult Honsha, and Sanofi and has received research funding from MSD, Daiichi Sankyo, and Ono. AT received a research grant from Takeda, MSD, LSK, and Sumitomo Dainippon. TKaj received honoraria from Taiho and Bayer. KYamaz received honoraria from Taiho and Bayer, and a research grant from Taiho. AM received honoraria from Lilly, Chugai and Takeda. TD received honoraria from SAWAI and Sysmex, and a research grant from MSD and ONO. NS received a research grant from MSD, Ono, Astellas, Dai-ichi-Sankyo. DS received honoraria from Chugai, and a research grant from Lilly, Ono, Dai-ichi-Sankyo, and Astellas, and endowed chairs from Yakult, Chugai, and Ono. MG received consultant fees from Dai-ichi-Sankyo, Ferring Pharmaceuticals, and Novartis. YS received a research grant from Taiho, MSD, and Lilly. The remaining authors declare that the research was conducted in the absence of any commercial or financial relationships that could be construed as a potential conflict of interest.

## Supplementary Material

Supplementary Figure 1 Kaplan–Meier curves of overall survival (OS) in the propensity score-matched dataset (cohort A vs cohort B). The median OS of cohort A and B were 9.3 months (95% CI, 8.2–10.5 months) and 5.3 months (95% CI, 4.8–6.7 months), respectively (*P* < 0.001).

Supplementary Table 1 Variables used for calculating propensity score.

Supplementary Table 2 Comparison of patient characteristics between regorafenib and TFTD groups in the propensity score-matched dataset.

Supplementary Table 3 Frequency of treatment-related grade≥3 AE in the propensity score-matched dataset.
