# Supplementary figures and images for: Survival Benefit of Crossover Administration of Regorafenib and Trifluridine/Tipiracil Hydrochloride for Patients With Metastatic Colorectal Cancer: Exploratory Analysis of a Japanese Society for Cancer of the Colon and Rectum Multicenter Observational Study (REGOTAS)
Source: Front Oncol. 2021 Mar 8;11:576036. doi: 10.3389/fonc.2021.576036 (PMC7982575; doi:10.3389/fonc.2021.576036)

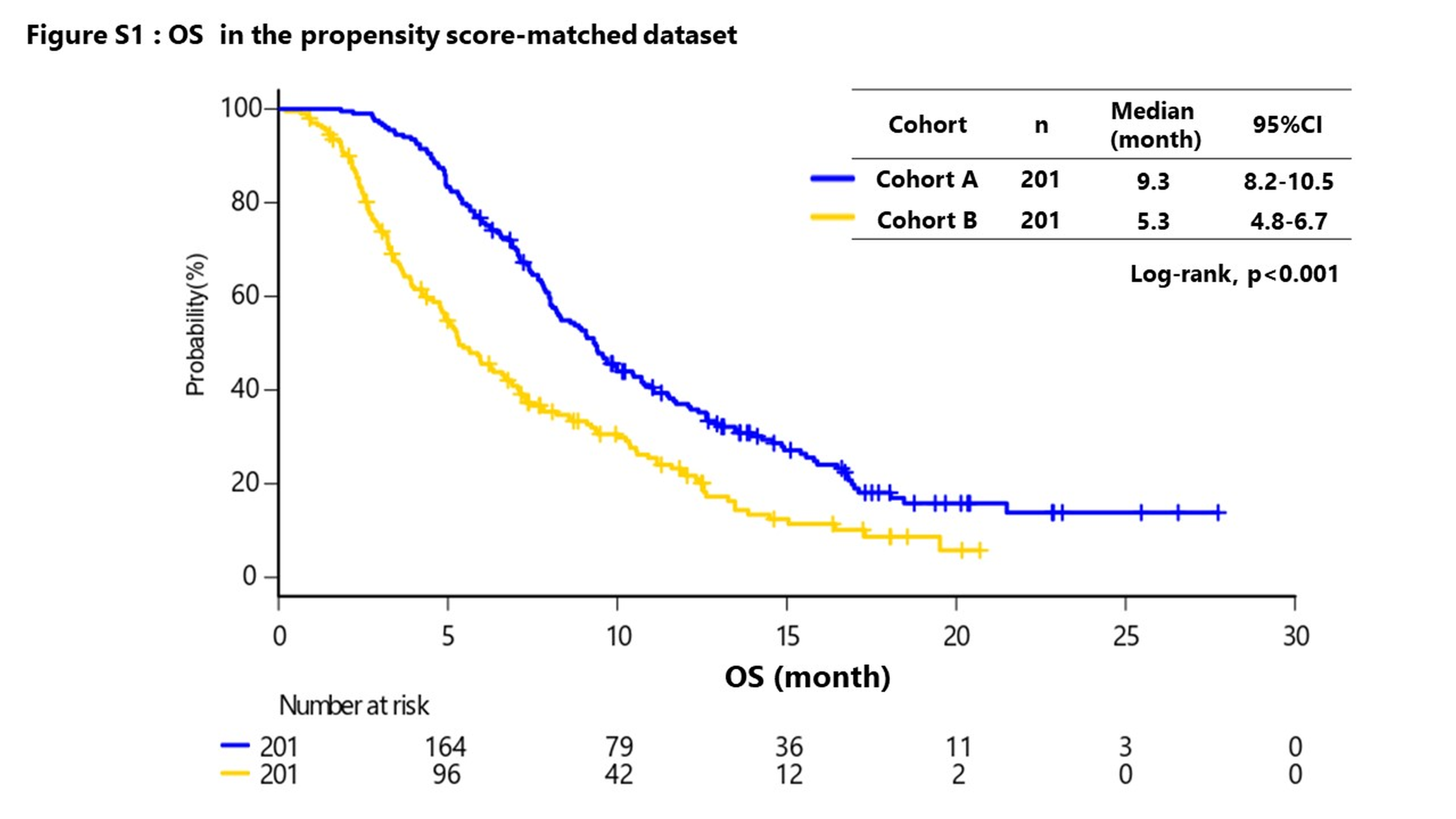

Supplement: Supplementary Figure 1 — Kaplan–Meier curves of overall survival (OS) in the propensity score-matched dataset (cohort A vs cohort B). The median OS of cohort A and B were 9.3 months (95% CI, 8.2–10.5 months) and 5.3 months (95% CI, 4.8–6.7 months), respectively (P < 0.001). [file Image_1.TIF]
