# Supplementary material for: Survival Benefit of Crossover Administration of Regorafenib and Trifluridine/Tipiracil Hydrochloride for Patients With Metastatic Colorectal Cancer: Exploratory Analysis of a Japanese Society for Cancer of the Colon and Rectum Multicenter Observational Study (REGOTAS)
Source: Front Oncol. 2021 Mar 8;11:576036. doi: 10.3389/fonc.2021.576036 (PMC7982575; doi:10.3389/fonc.2021.576036)
Supplement: Supplementary Table 1 — Variables used for calculating propensity score. [file Data_Sheet_1.docx]

**Supplementary Tables**

Supplementary Table S1. Variables used for calculating propensity score

| Variables | Category |
| --- | --- |
| Age | ≥65 vs <65 |
| Sex | Male vs Female |
| Body mass index | <18.5 kg/m^2^ vs ≥18.5 |
| EDOG PS | 1 or 2 vs 0 |
| Primary tumor site | left ^a^ vs right ^b^ |
| Surgery on primary tumor | Yes vs No |
| Pathologic type | Well/mod vs others |
| *RAS* status | MT vs WT |
| Liver metastasis | Yes vs No |
| Bone or peritoneal dissemination | Yes vs No |
| Number of metastatic organ(s) | ≥2 vs 1 |
| Intolerant to fluoropyrimidine | Yes vs No |
| Intolerant to oxaliplatin | Yes vs No |
| Intolerant to irinotecan | Yes vs No |
| Intolerant to bevacizumab | Yes vs No |
| Intolerant to anti-EGFR antibody | Yes vs No |
| Number of prior regimen | ≥3 vs <3 |
| Time since initiation of first-line chemotherapy | ≥18 months vs <18 |
| Baseline serum CEA value | ≥5 ng/ml vs <5 |

^a^ Including descending colon, sigmoid colon, and rectum.

^b^ Including cecum, ascending colon, and transverse colon.

^c^ Including *KRAS* or *NRAS* mutant-type.

^d^ Including *KRAS* exon 2 wild-type, if only the *KRAS* status was measured or wild-type *KRAS* exon 2-4 and wild-type *NRAS* exon 2-4, if both *KRAS* and *NRAS* status were measured.

Abbreviations: CEA, carcinoembryonic antigen; ECOG PS, Eastern Cooperative Oncology Group performance status; EGFR, epidermal growth factor receptor; *RAS*, rat sarcoma; WT, Wild-type; MT, Mutant

Supplementary Table S2. Comparison of patient characteristics between regorafenib and TFTD groups in the propensity score-matched dataset

|  | cohort A  n = 201 | | cohort B  n = 201 | | *P* value |
| --- | --- | --- | --- | --- | --- |
| **Age, year** |  |  |  |  |  |
| Median (IQR) | 64 | 56-70 | 64 | 57-71 | 0.668 |
| ≥65 years, n (%) | 93 | (46) | 99 | (49) | 0.618 |
| **Sex, n (%)** |  |  |  |  | 0.759 |
| Male | 125 | (62) | 121 | (60) |  |
| Female | 76 | (38) | 80 | (40) |  |
| **Body mass index, n (%)** |  |  |  |  | 0.404 |
| <18.5 kg/m^2^ | 34 | (17) | 27 | (13) |  |
| ≥18.5 kg/m^2^ | 167 | (83) | 176 | (87) |  |
| **ECOG PS, n (%)** |  |  |  |  | 0.708 |
| 0 | 88 | (44) | 80 | (40) | 0.479 |
| 1 or 2 | 113 | (56) | 121 | (60) |  |
| **Primary tumor site, n (%)** |  |  |  |  | 0.464 |
| right | 39 | (19) | 46 | (23) |  |
| left | 162 | (81) | 155 | (77) |  |
| **Surgery on primary tumor site, n (%)** |  |  |  |  |  |
| Yes | 160 | (80) | 159 | (79) | 1.000 |
| **Pathological type, n (%)** |  |  |  |  | 0.842 |
| Well-moderately differentiated adenocarcinoma | 184 | (92) | 182 | (91) |  |
| Others | 12 | (6) | 12 | (6) |  |
| Missing | 5 | (2) | 7 | (3) |  |
| ***RAS* status, n (%)** |  |  |  |  | 1.000 |
| Wild-type | 97 | (49) | 97 | (49) |  |
| Mutant | 101 | (50) | 101 | (50) |  |
| Missing | 3 | (1) | 3 | (1) |  |
| **Metastatic organ site, n (%)** |  |  |  |  |  |
| Liver | 126 | (63) | 130 | (65) | 0.756 |
| Lung | 135 | (67) | 123 | (61) | 0.253 |
| Lymph node | 78 | (39) | 92 | (46) | 0.189 |
| Peritoneal dissemination | 28 | (14) | 35 | (17) | 0.410 |
| Bone | 17 | (8) | 12 | (6) | 0.441 |
| **Number of metastatic organ site(s), n (%)** |  |  |  |  | 0.734 |
| 1 | 51 | (25) | 55 | (27) |  |
| ≥2 | 150 | (75) | 146 | (73) |  |
| **Intolerable drug, n (%)** |  |  |  |  |  |
| Any drugs | 54 | (27) | 63 | (31) | 0.380 |
| Fluoropyrimidine | 4 | (2) | 4 | (2) | 1.000 |
| Oxaliplatin | 51 | (25) | 47 | (23) | 0.727 |
| Irinotecan | 7 | (3) | 4 | (2) | 0.541 |
| Bevacizumab | 10 | (5) | 10 | (5) | 1.000 |
| Anti-EGFR antibody | 4 | (2) | 2 | (1) | 0.681 |
| **Prior regimen, n (%)** |  |  |  |  |  |
| ≥3 | 103 | (51) | 94 | (47) | 0.425 |
| **Time since initiation of first-line chemotherapy, n (%)** |  |  |  |  | 1.000 |
| <18 months | 49 | (24) | 48 | (24) |  |
| ≥18 months | 152 | (76) | 153 | (76) |  |
| **Baseline serum CEA, n (%)** |  |  |  |  |  |
| ≥5 ng/ml | 178 | (87) | 178 | (91) | 1.000 |

^＊^*P* values were calculated by Fisher’s exact probability test for categorical variables.

Abbreviations: IQR, interquartile range; EGFR, endothelial growth factor receptor; CEA, carcinoembryonic antigen; WT, Wild-type; MT, Mutant

Supplementary Table S3. Frequency of treatment-related grade≥3 AE in the propensity score-matched dataset.

| Variables | cohort A  (n = 201) |  | cohort B  (n = 201) |  | *P* value^＊^ |
| --- | --- | --- | --- | --- | --- |
| **Hematologic toxicities, n (%)** |  |  |  |  |  |
| Any | 56 | (28) | 57 | (28) | 1.000 |
| Neutropenia | 44 | (22) | 47 | (23) | 0.807 |
| Anemia | 12 | (6) | 18 | (9) | 0.343 |
| Thrombocytopenia | 7 | (3) | 7 | (3) | 1.000 |
| **Nonhematologic toxicities, n (%)** |  |  |  |  |  |
| Any | 49 | (24) | 47 | (23) | 0.907 |
| Fatigue | 3 | (1) | 5 | (2) | 0.721 |
| Anorexia | 4 | (2) | 12 | (6) | 0.074 |
| Febrile neutropenia | 3 | (1) | 4 | (2) | 1.000 |
| Hand-foot skin reaction | 21 | (10) | 6 | (3) | 0.005 |
| Liver dysfunction | 13 | (6) | 10 | (5) | 0.668 |

^＊^*P* values were calculated by Fisher’s exact probability test for categorical variables.

Abbreviations: AE, adverse event
